# Screening of the Toxicity of Polystyrene Nano- and Microplastics Alone and in Combination with Benzo(a)pyrene in Brine Shrimp Larvae and Zebrafish Embryos

**DOI:** 10.3390/nano12060941

**Published:** 2022-03-12

**Authors:** Ignacio Martínez-Álvarez, Karyn Le Menach, Marie-Hélène Devier, Miren P. Cajaraville, Hélène Budzinski, Amaia Orbea

**Affiliations:** 1 CBET Research Group, Department of Zoology and Animal Cell Biology, Research Centre for Experimental Marine Biology and Biotechnology PiE and Science and Technology Faculty, University of the Basque Country (UPV/EHU), E-48940 Leioa, Spain; ignacio.martinez@ehu.eus (I.M.-Á.); mirenp.cajaraville@ehu.eus (M.P.C.); 2 University of Bordeaux, UMR 5805 CNRS, EPOC, Laboratory of Physico- and Toxico-Chemistry of the Environment, CEDEX, F-33405 Talence, France; karyn.le-menach@u-bordeaux.fr (K.L.M.); marie-helene.devier@u-bordeaux.fr (M.-H.D.); helene.budzinski@u-bordeaux.fr (H.B.)

**Keywords:** polystyrene, nanoplastics, microplastics, benzo(a)pyrene, zebrafish embryos, brine shrimp larvae, acute toxicity, bioavailability

## Abstract

The occurrence of nanoplastics (NPs) and microplastics (MPs) in aquatic ecosystems and their capacity to sorb hydrophobic pollutants is nowadays an issue of great concern. This study aimed to assess the potential bioavailability and acute toxicity of polystyrene (PS) NPs (50 and 500 nm) and of MPs (4.5 µm), alone and with sorbed benzo(a)pyrene (B(a)P), in the embryo/larval stages of brine shrimps and zebrafish. Exposure to pristine plastics up to 50.1 mg PS/L did not cause significant impact on brine shrimp survival, while some treatments of plastics-B(a)P and all concentrations of B(a)P (0.1–10 mg/L) resulted acutely toxic. In zebrafish, only the highest concentrations of MPs-B(a)P and B(a)P caused a significant increase of malformation prevalence. Ingestion of NPs was observed by 24–48 h of exposure in the two organisms (from 0.069 to 6.87 mg PS/L). In brine shrimps, NPs were observed over the body surface and within the digestive tract, associated with feces. In zebrafish, NPs were localized in the eyes, yolk sac, and tail at 72 h, showing their capacity to translocate and spread into the embryo. MP ingestion was only demonstrated for brine shrimps. In zebrafish embryos exposed to plastics-B(a)P, B(a)P appeared in the yolk sac of the embryos. The presence of B(a)P was also noticeable in brine shrimps exposed to 500 nm NPs-B(a)P. In conclusion, NPs entered and spread into the zebrafish embryo and PS NPs, and MPs were successful vectors of B(a)P to brine shrimp and zebrafish embryos. Particle size played a significant role in explaining the toxicity of plastics–B(a)P. Our study provides support for the idea that plastics may pose a risk to aquatic organisms when combined with persistent organic pollutants such as B(a)P.

## 1. Introduction

Due to their durability and widespread use in populated areas, the presence of nano- (NPs) and microplastics (MPs) in aquatic ecosystems is reported worldwide [1]. The sources of NPs and MPs are numerous [2], with lighter NPs and MPs being more prone to arrive to aquatic ecosystems. The polymer types mostly present in aquatic ecosystems include polyethylene (PE), polypropylene (PP), polystyrene (PS), polyvinyl chloride (PVC), polyurethane (PUR), and polyethylene terephthalate (PET). PS has an intermediate density (1.05 g/cm^3^) among the previously mentioned polymers and is close to water density (1–1.03 g/cm^3^ depending on salinity). This makes PS MPs behave differently in waters of different salinity and become bioavailable for aquatic organisms, from surface water to bottom waters or sediments [3].

Several studies have already reported harmful effects of NPs and MPs on aquatic biota [4]. As reviewed for 28 taxonomic orders of zooplankton, MPs provoke negative effects on the feeding behavior, growth, development, and life span of some arthropods and fish larvae, with MP size and density being the main characteristics controlling their bioavailability [5]. Non-selective filter-feeders, such as brine shrimp, have been proposed as a suitable biological model in nanoecotoxicology, due to their cost-effectiveness [6], and standard guidelines, such as ISO/TS 20787 [7], have been developed to assess the toxicity of nanomaterials. Brine shrimps showed ingestion and elimination of NPs and MPs [8,9,10,11], which impaired food uptake. However, acute effects of PS MPs were not reported in brine shrimps. No significant mortality was observed in organisms exposed to up to 100 mg/L of 0.05, 0.1 or 10 µm- PS MPs for 24 or 48 h [8,9,12,13]. In contrast, exposure to PP MPs (size range 11.86–44.62 μm) caused oxidative stress and mortality in brine shrimp nauplii (LC50 40.947 μg/mL) [11].

Organisms such as fish that occupy a higher position in the trophic chain also ingest and accumulate MPs [14,15], the early life stages being especially sensitive to NP and MP impact [16]. Zebrafish embryos have been widely used to test the toxicity of dissolved chemicals and nanomaterials [17,18]. More recently, zebrafish embryos have also been utilized to test the toxicological effects of NPs and MPs [19,20,21,22,23]. Sub-lethal effects, such as changes in locomotor activity, heart beat rate, oxidative stress, and alterations in the nervous system, have been observed in zebrafish embryos 120 h post fertilization (hpf), after exposure to 0.1–10 mg/L of 0.025, 0.05, 0.1, or 45 µm PS MPs/NPs [10,22,23,24], and motility reduction has been recorded after exposure to 0.01–1 mg/L of PS MPs of 1 µm [19]. In addition to the impact of NPs and MPs, another concern has been raised regarding their ability to sorb other pollutants [25,26], especially persistent organic pollutants (POPs), featuring a high hydrophobicity and resistance to degradation (persistence) in the ecosystems. Most POPs are well-known toxic compounds to aquatic organisms, and many of them display carcinogenic properties [27]. This is the case of some of the ubiquitous polycyclic aromatic hydrocarbons (PAHs) [28]. Apart from polymer type, particle size is a key factor driving the sorption capacity of plastics; overall, the smaller the size of the plastics, the higher the sorption capacity [29]. Pollutants sorbed to NPs and MPs can be released inside organisms after assimilation [30].

Few studies have addressed the impacts produced by PS NPs/MPs combined with POPs in non-selective filter-feeders [29,31]. The potential transfer of C^14^ phenanthrene (0.05–1.2 mg/L) in co-exposure with two different sized PS particles (50 nm NPs and 10 µm MPs; 2.5–14.5 mg/L and 2.5–50 mg/L, respectively) was evaluated in *Daphnia magna*. The results showed that co-exposure for 2, 7, and 14 days with NPs provoked higher C14 phenanthrene bioaccumulation than co-exposure with MPs, due to the higher adsorption capacity of NPs for phenanthrene [29]. *D. magna* was also co-exposed to 100 nm PS NPs (0.01–75 mg/L) and polychlorinated biphenyls (PCBs, 0.640 mg/L). The increase in the PS NP concentration, combined with a fixed concentration of PCBs, increased the mortality of *D. magna*, indicating that NPs enhanced the PCB toxicity [31].

Fish larvae, including zebrafish embryos, have also been studied to evaluate the potential effect of PS NPs/MPs combined with POPs. Pannetier et al. [32] assessed the potential transport of B(a)P into medaka embryos, using MPs collected from beaches. A mixture with 1% of <600 µm MPs (PP, PE, and PS) coated with B(a)P (250 µg of B(a)P/g of MPs) caused high mortality (81%) of medaka embryos after 48 h of exposure, as well as an increase of 7-ethoxyresorufin-O-deethylase (EROD) activity. Smaller size MPs (200–250 µm, 400 mg/L) were also analyzed for their capacity to transport 17 α-ethynylestradiol (EE2, 0.001–1 µg/L) and phenanthrene (0.1–0.5 mg/L) into zebrafish embryos [33]. A decrease of EE2 and phenanthrene bioavailability, indicated by gene expression analysis in zebrafish embryos, was due to the sedimentation of MPs. When MPs and NPs were compared for potential transfer of compounds to zebrafish embryos, a higher EE2 bioaccumulation was observed in zebrafish co-exposed to 50 nm NPs (1 mg/L) and EE2 (2 and 20 µg/L) than in those co-exposed to 45 µm PS MPs (1 mg/L), which resulted in larval hypoactivity. This change in larval activity was not observed in embryos exposed to EE2 alone, indicating the capacity of NPs to enhance toxicity when combined with EE2 [20]. Higher bioaccumulation of phenanthrene was also observed in embryos co-exposed with small NPs (20 nm) than in embryos co-exposed with larger NPs (500 nm) or exposed to phenanthrene alone [34].While, 44 nm PS NPs (10 mg/L) alone and combined with PAHs from a sediment extract provoked a dysfunction of the mitochondrial function in zebrafish embryos. The reported mitochondrial dysfunction was no longer observed in the embryos exposed to the corresponding PAH mixture alone (5073 ng/mL), while cardiotoxicity and impaired brain vascularity were observed [35].

The aim of the present study was to test the potential bioavailability and acute toxicity of PS NPs and of MPs alone and with sorbed B(a)P, as a model pyrolytic PAH, in the embryo/larval stages of two model aquatic organisms, brine shrimps and zebrafish. Therefore, the specific objectives were (1) to assess the developmental toxicity of ‘pristine’ PS plastic particles of different sizes (50 nm, 500 nm, and 4.5 µm) and of dissolved B(a)P on brine shrimp larvae and zebrafish embryos; (2) to assess the toxicity of NPs (500 nm) and MPs (4.5 µm) with sorbed B(a)P; and (3) to evaluate the availability of NPs and of MPs with sorbed B(a)P to brine shrimp larvae and zebrafish embryos. As the aim of the study was to test the accumulation and effects of B(a)P sorbed to the plastics, avoiding co-exposure to dissolved B(a)P and plastics, particles were filtered after incubation with B(a)P. Owing to this, 50 nm NPs were not combined with B(a)P, due to the technical difficulty of filtering and efficiently recovering 50 nm NPs after incubation, as was done for the other particle sizes.

## 2. Materials and Methods

### 2.1. Chemicals and Plastic Particles

Benzo(a)pyrene (B(a)P, purity ≥ 96%), benzo(a)pyrene d_12_ (B(a)P d_12_, ≥ 98%), and dimethylsulfoxide (DMSO, purity ≥ 96%) were purchased from Sigma-Aldrich (St. Louis, MO, USA). Fluorescent Fluoresbrite^®^ carboxylate 50 nm PS NPs (excitation/emission wavelengths of 360/407 nm), non-fluorescent PS NPs (50 and 500 nm), and MPs (4.5 µm) in aqueous suspensions were purchased from Polysciences Inc. (Warrington, PA, USA). The concentration of the commercial stocks was 2.5% (3.64 × 10^14^ particles/mL for 50 nm NPs, 3.64 × 10^11^ particles/mL for 500 nm NPs and 4.99 × 10^8^ particles/mL for 4.5 µm MPs).

### 2.2. Preparation and Analysis of Exposure Media

B(a)P concentrations used for toxicity assays were 0.1, 0.5, and 1 mg/L B(a)P in 0.01% DMSO and 5 and 10 mg/L B(a)P in 0.1% DMSO. An initial stock solution of 10 g/L (50 mg of B(a)P in 5 mL pure DMSO) was prepared and used to prepare the other two stock solutions of 5 and 1 g/L of B(a)P in 100% DMSO. These stock and intermediate solutions were stored at −20 °C in closed glass vials. All the stock solutions, intermediate solutions, and final dilutions were dispersed for 10 min in an ultrasounds bath (VWR, Radnor, PA, USA) before being used. Each stock solution was diluted 1:1000 in the exposure medium required for each experiment (MilliQ water for chemical analysis of B(a)P concentration, embryo water for zebrafish embryo toxicity test or salt water for brine shrimp immobilization test), in order to obtain the three highest B(a)P exposure concentrations (10, 5, and 1 mg/L B(a)P) in 0.1% (*v*/*v*) DMSO). Then, 0.5 and 0.1 mg/L B(a)P were prepared in 0.01% DMSO by diluting the previous ones 1:10 in exposure media.

Immediately after being prepared, an aliquot from each concentration was collected for chemical analysis and diluted with MilliQ water up to 1 µg/L in 10 mL glass vials. Perdeuterated B(a)P was added as an internal standard prior the analysis, in order to per-form quantification by isotopic dilution. Analysis was performed by gas chromatography and mass spectrometry (GC/MS) after solid phase microextraction (SPME), using selected ion monitoring mode (electronic impact ionization). SPME consisted in a heating process at 40 °C with 35 min stirring period at 250 rpm of the polydimethylsiloxane (PDMS) fiber (Supelco, Sigma-Aldrich, South Africa). The fiber (100 µm) was thermally desorbed into the GC/MS system (Agilent GC 7890A/Agilent MSD 5975C, Agilent Technologies, Santa Clara, CA, USA) for 10 min at 280 °C. The GC/MS system was operated with an energy of ionization of 70 eV in ionization mode for electronic impact equipped with an HP5MS-UI column (5% phenyl methylpolysiloxane; 30 m × 0.25 mm i.d.; 0.25 μm sorbent, Agilent Technologies). Injection in the GC/MS system was done with the inlet temperature at 280 °C, in the pulsed splitless mode; a pulse pressure of 25 psi was maintained for 1.5 min, the purge flow to split vent was 60 mL/min after 1.5 min. The injection volume was 1 μL, with a helium (purity 6.0) constant flow rate of 1.3 mL/min. The column temperature was initially held at 50 °C for 2 min, and was then increased to 250 °C at 10 °C/min for 1 min, to 280 °C for 2 min and to 310 °C at 10 °C/min, where it was held for 3 min. Compound determination was operated in the selected ion monitoring (SIM). B(a)P was quantified by isotope dilution (B(a)P d_12_).

In order to assess whether there was any B(a)P loss during experimental exposures, 2 mL of each concentration were placed in duplicate in a 24-well microplate. The microplate was maintained under a photoperiod simulating the test conditions (12 h light/12 h dark, 20 °C). Aliquots were taken at 24, 48, and 120 h, diluted up to 1 µg/L, and processed as described before.

Exposure concentrations for PS NPs and MPs of the three sizes, in terms of number of particles and in terms of PS mass used in the toxicity assays, are shown in Table 1. To prepare 500 nm PS NPs and 0.45 µm PS MPs with sorbed B(a)P, 50 mg PS/L was incubated in glass bottles with 100 µg/L B(a)P (in 0.01% DMSO) prepared in MilliQ water. The incubation volume used for MPs contamination with B(a)P depended on the amount of MPs required for the assays, always keeping the PS concentration used in previous sorption experiments (50 mg/L). PS suspensions were wrapped with aluminum and shaken at 300 rpm for 24 h at 20 °C. Then, samples were filtered through a polyethersulfone filter (0.45-µm filter pore, Sarstedt AG & Co., Nümbrecht, Germany) and washed two times with 10 mL of MilliQ water. PS particles were recovered from the filters using salt water or embryo water for brine shrimps or zebrafish, respectively. These NPs-B(a)P and MPs-B(a)P suspensions were diluted with the corresponding medium to prepare the five exposure concentrations (Table 1). The recovery of 4.5 µm MPs after filtration was measured using a Coulter Counter (Z-counter, Beckman Coulter, Brea, CA, USA) (Figure S1).

### 2.3. Acute Toxicity Assays

#### 2.3.1. Brine Shrimp Cultures and Immobilization Test

One gram of brine shrimp cysts (Artemia Koral GmbH, Nürnberg, Germany) was incubated with vigorous aeration in a temperature-controlled room at 26 °C under continuous illumination in 30‰ salt water prepared from commercial salt (Sera, Heinsberg, Germany). Hatched larvae were harvested with a glass pipette, avoiding collecting unhatched cysts, and transferred to a freshly prepared salt medium, where larvae were maintained for 24 or 48 h, before starting the toxicity tests. At 24 or 48 h post hatch (hph), cultures were moved for some hours to a temperature-controlled room at 20 °C to acclimate individuals for the toxicity assays, which were performed following the procedure previously described in Lacave et al. [36], using immobilization as a criterion for acute toxicity. The exposures to non-fluorescent PS NPs and MPs were run in covered 24-well microplates at 20 ± 1 °C, with a photoperiod of 12 h light/12 h dark. Five brine shrimps were placed in each well with 2 mL of exposure medium, and 6 wells were used per concentration (30 individuals per experimental group and 30 individuals as controls). The selection of individuals was made under a stereoscopic microscope (Nikon smz800, Kanagawa, Japan) paying attention to the morphology corresponding to the 24 hph and 48 hph stages. After 24 and 48 h of exposure, the amount of immobilized larvae was recorded. A larva was considered as immobile when it was not able to move after shaking the plate. The toxicity of 0.01, 0.05, and 0.1% DMSO was tested in parallel.

#### 2.3.2. Zebrafish Maintenance, Egg Production, and Embryo Toxicity Test

The zebrafish (wild type AB Tübingen) stock was maintained in a temperature-controlled room at 28 °C with a 14 h light/10 h dark cycle in 100 L tanks provided with mechanical and biological filters, following standard protocols for zebrafish culture. Conditioned water (600 µS/cm and 7–7.5 pH) was prepared from deionized water and commercial salt. Fish were fed twice per day with Vipagran baby (Sera, Heinsberg, Germany) and brine shrimp nauplii of 24 hph, cultured as described above. Breeding female fish were selected and maintained separately in fish breeding nets inside the same tanks, in order to avoid continuous spawning. The day before the assay, one female and two male zebrafish were placed separately in a breeding trap, previously located in a 2-L tank containing conditioned water. Fish were left overnight and, just before the light was switched on, the separation was removed. The fertilized eggs were collected in a Petri dish with the help of a Pasteur pipette. The eggs selected as viable under a Nikon smz800 (Nikon, Kanagawa, Japan) stereoscopic microscope were transferred to the exposure microplates.

The toxicity tests were carried out in covered 24-well microplates, placing one embryo per well in 2 mL of test solution made in embryo water (600–800 µS/cm, 6.5–6.8 pH) [37] following the OECD guideline TG236 [38]. In each microplate, two different concentrations were tested (10 embryos per concentration). Four control embryos were placed in embryo water in the remaining wells. For each exposure condition, three microplates were prepared, resulting in 30 embryos exposed to each concentration and 36 control embryos. Newly fertilized embryos (<2 hpf) were exposed to the non-fluorescent PS NPs and MPs up to 120 hpf. The test was only considered valid when the survival of the control group of each replicate was ≥90%. The toxicity of 0.01, 0.05, and 0.1% DMSO was tested in parallel. Daily and up to the end of the test, embryos were examined to determine survival rate (as the percentage of alive embryos at 120 hpf), hatching time (as the time that embryos need to hatch), and malformation prevalence (as the percentage of malformed embryos over surviving embryos at 120 h). Normal embryo morphology and malformations were based on Fako and Furgeson [39]. Developmental abnormalities scored as malformations were spinal cord flexure, caudal fin alteration, tail malformation, pericardial edema, yolk sac edema, eye abnormality, and stunted body. Malformations were recorded and photographed under a Nikon AZ100 stereoscopic microscope.

### 2.4. Analysis of Bioavailability of Plastic Particles and B(a)P

Distribution of fluorescent 50 nm PS NPs alone, and of non-fluorescent 500 nm PS NPs and 4.5 µm MPs with sorbed B(a)P, within brine shrimp larvae and developing zebrafish embryos was examined under a confocal microscope (Olympus Fluoview FV500, Tokyo, Japan). All the individuals used for these analyses were exposed at the same time as those used for toxicity assays and at the same exposure concentrations. Transmitted and fluorescence confocal images of Z-stacks series were acquired using a 10x UPLAPO NA0.45 lens. Fluorescence images were obtained by excitation at 405 nm and emission at 430–460 nm. Fluorescence emitted by B(a)P accumulated in brine shrimp larvae and zebrafish embryos exposed to B(a)P alone was analyzed at the end of the toxicity assays using a Cytation 5 microplate reader (Biotek Instruments Inc., Winooski, VT, USA) provided with a blue filter (360/460 nm excitation/emission wavelengths). For imaging, brine shrimp larvae and zebrafish embryos were anesthetized with 2% (*v*/*v*) chloroform in salt water and with 200 mg/L benzocaine prepared in embryo water, respectively.

### 2.5. Statistical Analyses

Data on survival of brine shrimp larvae and of zebrafish embryos and prevalence of malformations in zebrafish embryos were analyzed by binomial logistic regression [36,40]. Odd ratios were calculated, in order to estimate and compare the risk associated with the exposures. EC_50_ and LC_50_ values with confidence intervals (CI) of 5 to 95% were calculated using a Probit model using the R package (R Foundation for Statistical Computing, Vienna, Austria). Hatching time of zebrafish embryos was tested for normality (Kolmogorov-Smirnov test) and homogeneity of variances (Bartlett test). Then, data were analyzed by one-way ANOVA, followed by the Tukey post hoc test (*p* < 0.05), using the GraphPad Prism version 5.00 for Windows (GraphPad Software, La Jolla, CA, USA).

## 3. Results

### 3.1. Analysis of the B(a)P Exposure Media

Measured B(a)P concentrations over time for the five exposure concentrations are shown in Table 2. B(a)P concentration measured at 0 h was always lower (29–63%) than the nominal concentration. B(a)P concentration was constant during the first 24 h, with the exception of 5 mg/L of B(a)P, whose concentration dropped from 1.496 ± 0.707 at 0 h to 1.173 ± 0.505 mg/L at 24 h. At 48 h, the B(a)P concentrations dropped in all cases, except for 10 mg/L B(a)P, which remained quite stable until 120 h. At this time, the lowest B(a)P concentration (0.1 mg/L) presented the highest relative loss (up to 48.3% of the initially measured concentration). Intermediate concentrations showed similar or even higher values than those measured at 48 h.

### 3.2. Toxicity Assays in Brine Shrimp Larvae

Effect on survival of the exposure to NPs and to MPs alone and with sorbed B(a)P for 24 h and 48 h of 24 hph and 48 hph brine shrimp larvae is shown in Table 3. Larvae exposed to pristine NPs and MPs did not show any significant difference on survival compared to controls at any exposure concentration or time. Survival rate of exposed larvae ranged from 75% in the exposure of 48 hph larvae for 48 h, to 50.1 mg/L of 500 nm NPs to 100%. Exposure of 24 hph larvae to 0.00034 mg/L, 0.00069 mg/L, and 6.87 mg/L of 500 nm NPs-B(a)P for 48 h caused a significant decrease in survival rate compared to control larvae. The 48 hph larvae exposed to 0.00034 mg/L of 500 nm NPs-B(a)P for 48 h also showed a significant reduction in survival compared to control larvae. B(a)P alone resulted the most toxic treatment, except for 24 hph brine shrimp larvae exposed for 24 h, which did not show any detrimental effect. However, exposure for 48 h to all B(a)P concentrations provoked a significant reduction in survival compared to control larvae, declining up to 59.4% in larvae exposed to the highest concentration. In the case of 48 hph larvae, exposure to any B(a)P concentration for 24 or 48 h provoked 100% mortality. Exposure of brine shrimp larvae of 24 hph or 48 hph up to 0.1% DMSO for 24 h or 48 h did not provoke any acute toxic effect (Table S1).

In the case of brine shrimp larvae of 24 hph exposed for 48 h to 500 nm NPs-B(a)P, estimated LC_50_ value was 4.75 ± 1.03 mg/L (95% CI: 2.7–6.81). For the other treatments with plastic particles, LC_50_ values were always higher than the highest tested concentration. For B(a)P alone, calculated LC_50_ values for brine shrimp larvae of 24 hph were also higher than the highest tested concentration (nominal concentration 10 mg B(a)P/L, measured initial concentration 4.92 mg/L), while for brine shrimp larvae of 48 hph LC_50_ values of 0.004 ± 0.197 mg B(a)P/L (95% CI: −0.390–0.397) and 0.002 ± 0.119 mg B(a)P/L (95% CI: −0.236–0.240) for 24 h and 48 h of exposure, respectively, were calculated based on the measured concentrations.

Comparison between exposure treatments revealed some significant effects of the sorbed B(a)P and of the plastic particle size in the survival rate of brine shrimp larvae. Regarding the effect of the sorbed B(a)P, exposure of 24 hph larvae to 0.00069 mg/L and 6.87 mg/L of 500 nm NPs-B(a)P for 48 h caused a significant decrease in survival compared to the exposure to 500 nm NPs alone (Table 3, Table S2). Similarly, exposure of 48 hph larvae to 0.00034 mg/L, 0.687 and 6.87 mg/L of 500 nm NPs-B(a)P for 48 h caused a significant decrease in survival, compared to the exposure to 500 nm NPs alone. Significant differences were observed in survival rate of 24 hph larvae exposed for 48 h to 0.025 mg/L, 0.501 mg/L and 50.1 mg/L of 4.5 µm MPs-B(a)P, compared to larvae exposed to the same concentration of 4.5 µm MPs alone. Regarding the effect of the particle size, in the case of plastic particles with sorbed B(a)P, 500 nm NPs had a stronger effect than 4.5 µm MPs, the difference being statistically significant in the case of 24 hph larvae exposed to 6.87 mg/L of 500 nm NPs-B(a)P, and for 48 hph brine shrimp larvae exposed to 6.87 and 0.687 mg/L of 500 nm NPs-B(a)P for 48 h.

### 3.3. Toxicity Assays in Zebrafish Embryos

The results of the developmental parameters of zebrafish embryos are presented in Table 4 and Table S3. Exposure to PS NPs and to MPs alone or in combination with B(a)P did not provoke any significant effect on zebrafish embryo survival at 120 hpf or in hatching time. Only the exposure of embryos to 50.1 mg/L of 4.5 µm MPs-B(a)P caused a significant increase (up to 56.7%) of malformed embryos at 120 hpf. A similar prevalence of malformed embryos was recorded after exposure to 5 mg/L (50%) and 10 mg/L (58.6%) of B(a)P alone. Exposure to B(a)P caused a concentration-dependent increase of malformation prevalence. Previous toxicity assays with different DMSO concentrations showed that the DMSO concentration present in the B(a)P exposure was not deleterious for zebrafish embryos (Table S4). The EC_50_ values estimated for malformation prevalence at 120 hpf of embryos exposed to NPs and to MPs alone and to 500 nm NPs-B(a)P were always above the highest exposure concentration. EC_50_ values calculated for embryos exposed to 4.5 µm MPs-B(a)P and to B(a)P alone were 45.57 ± 9.12 mg/L (95% CI: 27.34–63.81) and 3.55 ± 0.68 mg/L (95% CI: 2.183–4.915), respectively.

**Figure 1:**
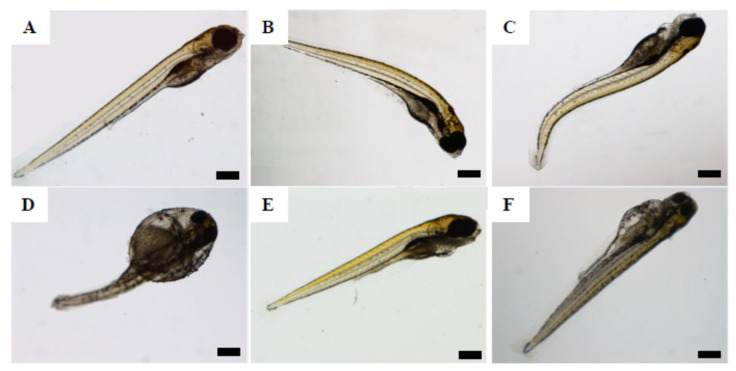
Malformations found in exposed zebrafish embryos at 120 hpf. (**A**) unexposed control embryo showing normal morphology; (**B**) embryo exposed to 0.687 mg/L of 500 nm NPs showing spinal cord flexure; (**C**) embryo exposed to 0.069 mg/L of 500 nm NPs-B(a)P showing pericardial edema and spinal cord flexure; (**D**) embryo exposed to 50.1 mg/L of 4.5 µm MPs-B(a)P showing multiple malformations, such as pericardial edema, yolk sac edema, and spinal cord flexure; (**E**) embryo exposed to 10 mg/L of B(a)P showing pericardial edema; (**F**) embryo exposed to 1 mg/L of B(a)P showing yolk sac edema. Scale bars: 100 µm.

The prevalence of individual malformations is also shown in Table 4, and Figure 1 illustrates some of the malformations found. Spinal cord flexure was the most prevalent malformation found in embryos exposed to all the treatments (Figure 1B–D). Those exposed to 4.5 µm MPs-B(a)P (Figure 1D) and to B(a)P presented the highest prevalence, with values of 36.7% and 44.8%, respectively. High prevalence of pericardial edema (24.1% and 30%, respectively) was observed in embryos exposed to the highest concentrations of B(a)P alone (Figure 1E) or 4.5 µm MPs-B(a)P (Figure 1D). Yolk sac edema (Figure 1C) was only observed in one or two individuals per concentration and treatment, such as in embryos exposed to 5.1 and 50.1 mg/L of 4.5 µm MPs-B(a)P (3.3% and 6.7%, respectively), and to 1 and 5 mg/L of B(a)P (3.8% and 3.4%, respectively) (Figure 1D,F). Only one case of eye abnormality was observed in an embryo exposed to 0.687 mg/L of 500 nm NPs.

### 3.4. Bioavailability of Plastic Particles and B(a)P

#### 3.4.1. Brine Shrimp Larvae

**Figure 2:**
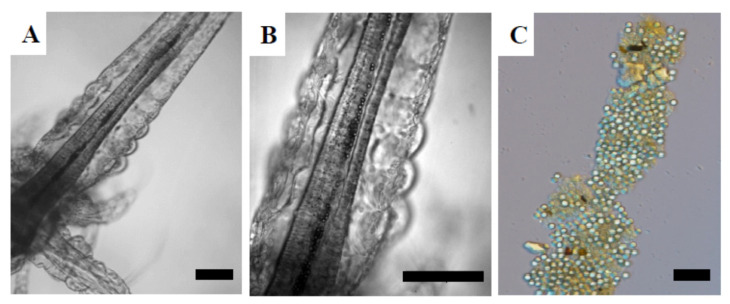
Brine shrimp larvae exposed to 4.5 µm MPs. (**A**) 24 hph larva exposed to 50.1 mg/L for 48 h; (**B**) 4.5 µm MPs in the digestive tract of the larva shown in (**A**) at higher magnification; (**C**) feces of brine shrimp larvae exposed to 50.1 mg/L. Scale bars: 100 µm (**A**,**B**) and 25 µm (**C**).

Using stereoscopic microscopy, only ingestion of 4.5 µm MPs was observed in brine shrimp larvae. Figure 2 shows MPs in the digestive tract of 24 hph larvae exposed for 48 h to 50.1 mg/L (Figure 2A,B). No evidence of MP internalization into the tissues was found. While, 4.5 µm MPs were also found in 48 hph brine shrimp feces (Figure 2C) after 24 h of exposure. Due to their small size, 50 nm and 500 nm NPs could not be identified under the stereoscopic microscope.

**Figure 3:**
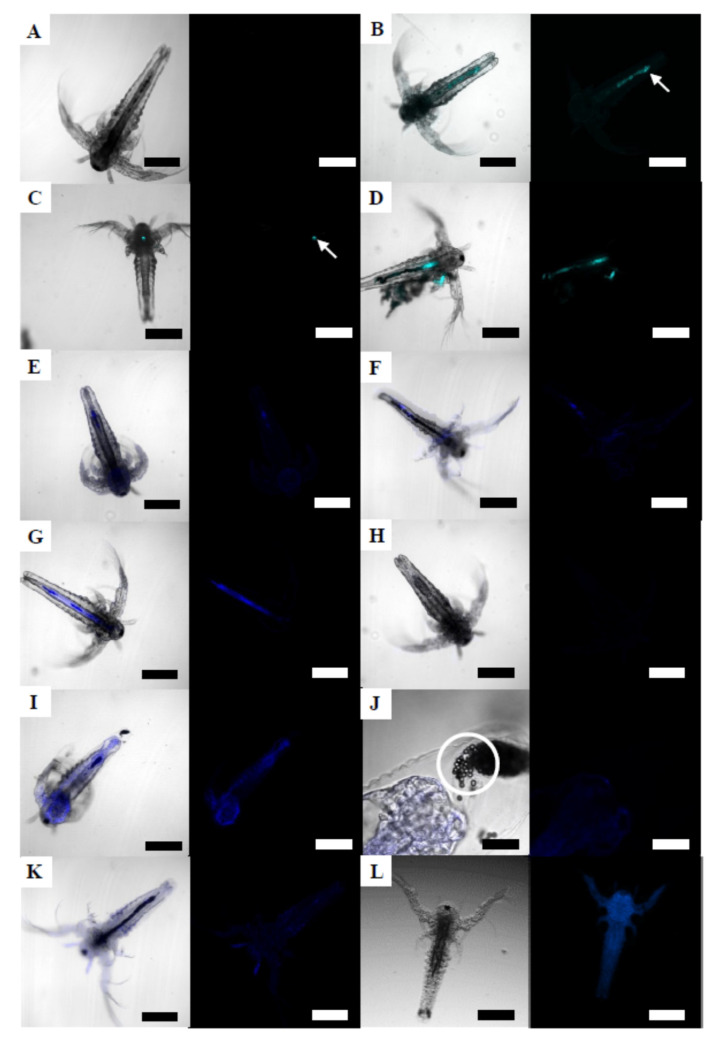
Confocal micrographs of brine shrimp larvae exposed to 50 nm NPs and to MPs with sorbed B(a)P (**A**–**K**) and Cytation 5 micrographs of larvae exposed to B(a)P alone (**L**). A) unexposed control larva at 72 hph; (**B**) 24 hph larva exposed to 6.87 mg/L of fluorescent NPs for 24 h, showing fluorescence along the digestive tract and in the feces (white arrow); (**C**) 24 hph larva exposed to 6.87 mg/L of fluorescent NPs for 48 h, showing fluorescence in the labrum (white arrow); (**D**) 48 hph larva exposed to 6.87 mg/L of NPs for 24 h showing fluorescence inside the digestive tract; (**E**) 24 hph larva exposed for 24 h to 6.87 mg/L of 500 nm NPs-B(a)P; (**F**) 24 hph larva exposed for 48 h to 0.00069 mg/L of 0500 nm NPs-B(a)P; (**G**) 48 hph larva exposed for 24 h to 6.87 mg/L of 500 nm NPs-B(a)P; (**H**) 24 hph larva exposed for 24 h to 50.1 mg/L of 4.5 µm MPs-B(a)P; (**I**) 24 hph larva exposed for 48 h to 50.1 mg/L of 4.5 µm MPs-B(a)P; (**J**) 4.5 µm MPs-B(a)P (white circle) excreted from the digestive tract of a 24 hph larva exposed for 48 h observed at higher magnification; (**K**) 48 hph larvae exposed for 48 h to 5.01 mg/L of 4.5 µm MPs-B(a)P; (**L**) 48 hph larvae exposed for 48 h to 0.1 mg/L of B(a)P. Scale bars: 200 µm (**A**–**I**,**K**,**L**) and 50 µm (**J**).

The bioavailability of fluorescent 50 nm NPs, of 500 nm NPs and 4.5 µm MPs with sorbed B(a)P was studied by confocal microscopy (Figure 3). Unexposed brine shrimp did not show any fluorescence signals at any developmental stage (Figure 3A). Regarding 50 nm NPs, individual NPs could not be identified, due to the resolution of the microscope, but fluorescence indicating the presence of particles was detected, mainly along the digestive tract, in the feces (Figure 3B,D), and in the labrum (Figure 3C) at two NPs concentrations (0.687 and 6.87 mg/L) and in brine shrimps of both stages (24 hph and 48 hph).

At early stages, 24 hph larvae exposed to 6.87 mg/L of 500 nm NPs-B(a)P for 24 h showed fluorescence all over the body and inside the digestive tract (Figure 3E). The 24 hph larvae exposed to 0.00069 mg/L (the second lowest concentration) also presented fluorescence (Figure 3F). In brine shrimps of 48 hph, fluorescence inside the digestive tract was again observed after exposure to concentrations between 0.00069 mg/L and 6.87 mg/L of 500 nm NPs-B(a)P for 24 h (Figure 3G). After 48 h of exposure, brine shrimp larvae did not present fluorescence at any exposure concentration. No fluorescence signal was detected in 24 hph brine shrimp larvae exposed to any concentration of 4.5 µm MPs-B(a)P for 24 h (Figure 3H), but after 48 h of exposure to 50.1 mg/L, fluorescence was observed all over and inside (digestive tract) the brine shrimp body (Figure 3I). As shown in the micrograph of the same larvae taken at a higher magnification (Figure 3J), the digestive tract was full of 4.5 µm MPs that can be seen individually. The fluorescence indicating the presence of B(a)P appeared in the surface and inside of larvae, but no fluorescence was observed on the surface of the ingested 4.5 µm MPs-B(a)P. At longer exposure times, brine shrimp larvae exposed to 4.5 µm MPs-B(a)P presented fluorescence on the surface of the body at exposure concentrations above 5.01 mg/L (Figure 3K). As previously mentioned, B(a)P emitted fluorescence, which was clearly observed using the Cytation 5 microplate reader in 48 hph brine shrimp larvae exposed to dissolved B(a)P for 48 h (100 µg/L; Figure 3L).

#### 3.4.2. Zebrafish Embryos

**Figure 4:**
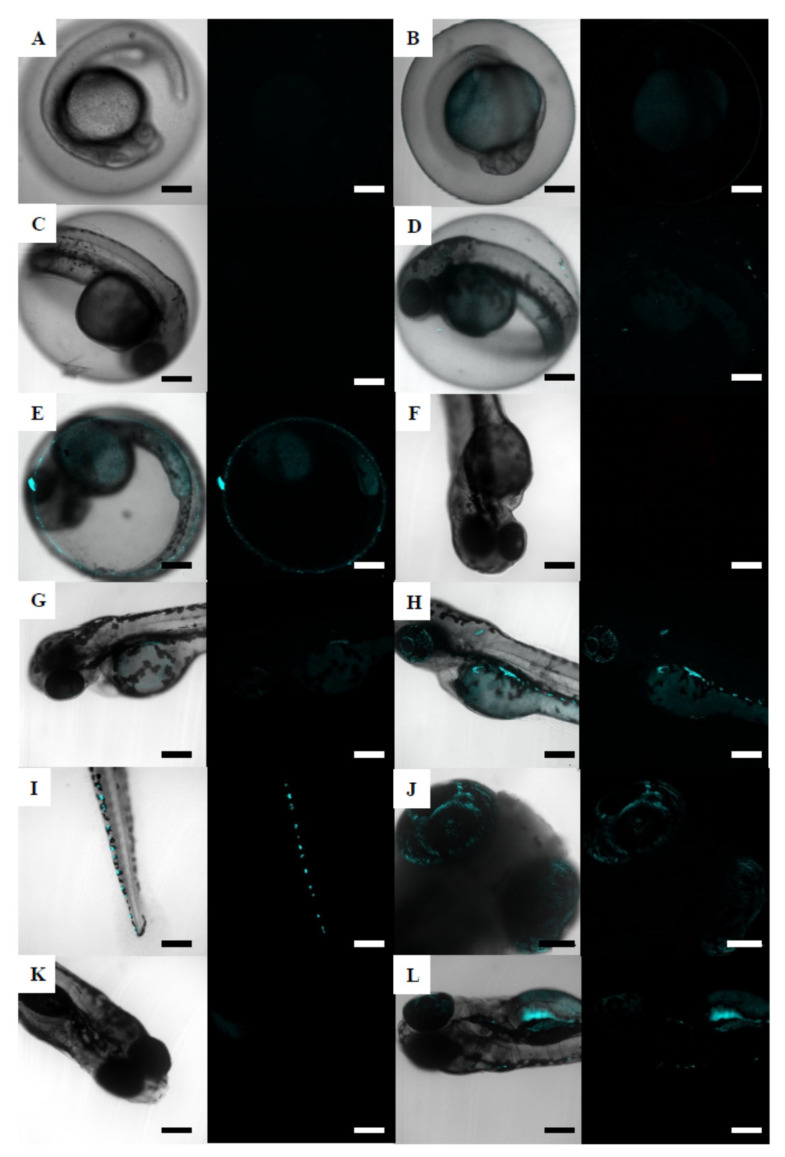
Confocal micrograph stacks of zebrafish embryos exposed to fluorescent 50 nm NPs. (**A**) unexposed control embryo at 24 hpf; (**B**) 24 hpf embryo exposed to 6.87 mg/L NPs; (**C**) unexposed control embryo at 48 hpf; (**D**) 48 hpf embryo exposed to 0.687 mg/L NPs; (**E**) 48 hpf embryo exposed to 6.87 mg/L NPs; (**F**) unexposed control embryo at 72 hpf; (**G**) 72 hpf embryo exposed to 0.069 mg/L NPs; (**H**) 96 hpf embryo exposed to 6.87 mg/L NPs; (**I**) 96 hpf embryo exposed to 6.87 mg/L; (**J**) 96 hpf embryo exposed to 6.87 mg/L; (**K**) unexposed control embryo at120 hpf; (**L**) 120 hpf embryo exposed to 0.069 mg/L. Scale bars: 200 µm (**A**–**I**,**K**,**L**) and 100 µm (**J**).

As mentioned for brine shrimps, individual fluorescent 50 nm NPs could not be detected in the embryos, due to the lack of resolution of the confocal microscope for this particle size. However, a fluorescence signal indicating the presence of NPs was found distributed in different organs of the embryo: eye, tail, and yolk sac (Figure 4). For the earliest stages, zebrafish embryos of 24 hpf showed fluorescence on the chorion surface and in the yolk sac (Figure 4B), only at the highest exposure concentration (6.87 mg/L). At 48 hpf, fluorescence appeared in embryos exposed to 0.687 mg/L (Figure 4D) and was increased in embryos exposed to 6.87 mg/L (Figure 4E). The lowest exposure concentration at which fluorescence was detected in 72 hpf embryos was 0.069 mg/L (Figure 4G). Hatched embryos of 72 hpf showed fluorescence in the yolk sac (Figure 4H), in discrete areas near the yolk sac and along the tail (Figure 4I), and within the eyes (Figure 4J). At the end of exposure (120 h), fluorescence remained as shown for 72 hpf exposed embryos, and it also appeared in embryos exposed to a lower concentration (0.069 mg/L, Figure 4L). Unexposed zebrafish embryos did not show fluorescence at any stage (Figure 4A,C,F,K).

**Figure 5:**
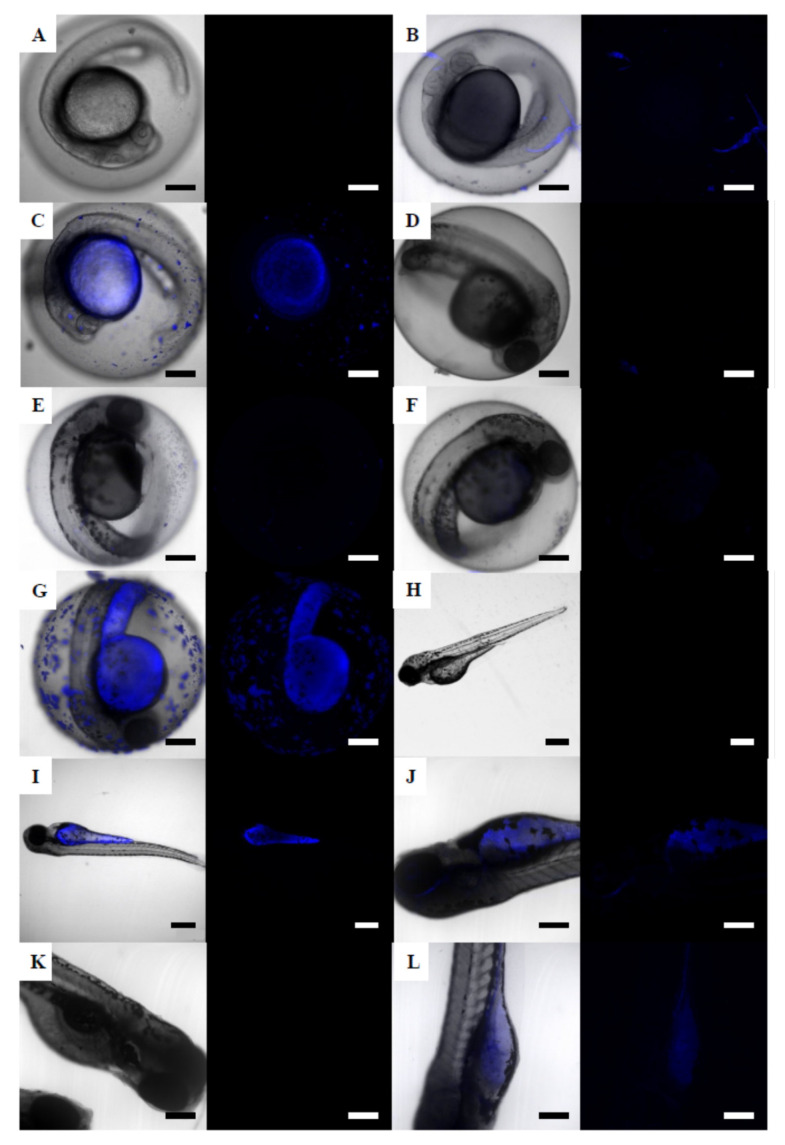
Confocal micrograph stacks of zebrafish embryos at different stages of development exposed to 500 nm NPs-B(a)P. (**A**) 24 hpf unexposed control embryo; (**B**) 24 hpf embryo exposed to 0.687 mg/L; (**C**) 24 hpf embryo exposed to 6.87 mg/L; (**D**) 48 hpf unexposed control embryo; (**E**) 48 hpf embryo exposed to 0.687 mg/L; (**F**) 48 hpf embryo exposed to 0.687 mg/L; (**G**) 48 hpf embryo exposed to 6.87 mg/L; (**H**) 72 hpf unexposed control embryo; (**I**) 72 hpf embryo exposed to 6.87 mg/L; (**J**) 96 hpf embryo exposed to 0.687 mg/L; (**K**) 120 hpf unexposed control embryo; (**L**) 120 hpf embryo exposed to 0.069 mg/L. Scale bars: 200 µm (**A**–**G**,**J**–**L**) and 300 µm (**H**,**I**).

**Figure 6:**
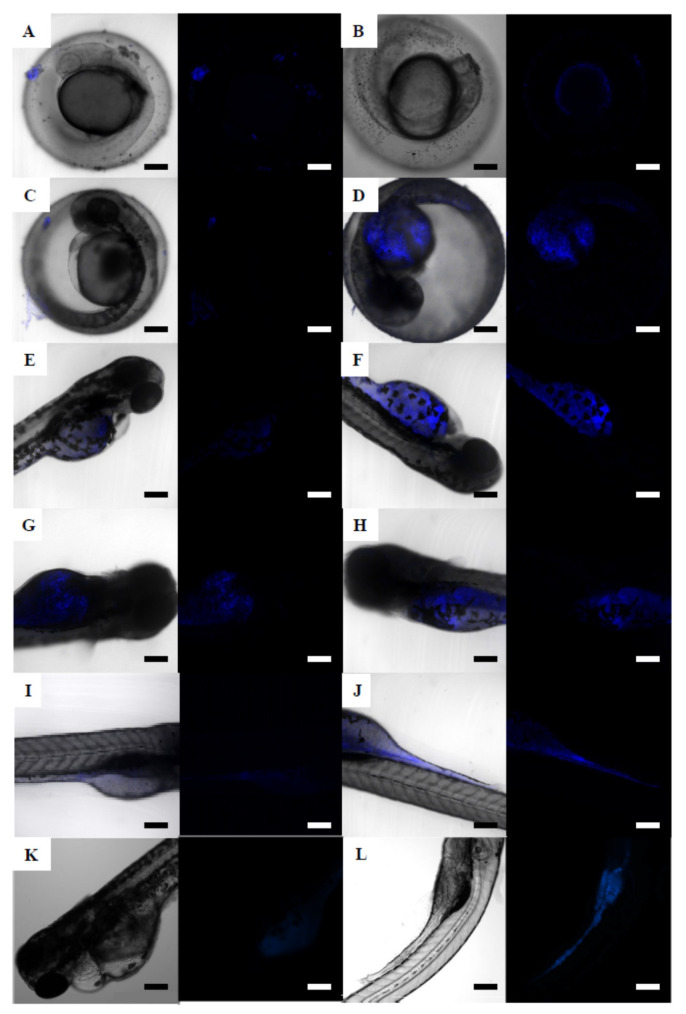
Confocal micrograph stacks of zebrafish embryos at different stages of development exposed to 4.5 µm MPs-B(a)P (**A**–**J**), and Cytation 5 micrographs of embryos exposed to B(a)P alone (K-L). (**A**) 24 hpf embryo exposed to 5.01 mg/L; (**B**) 24 hpf embryo exposed to 50.1 mg/L; (**C**) 48 hpf embryo exposed to 5.01 mg/L; (**D**) 48 hpf embryo exposed to 50.1 mg/L; (**E**) 72 hpf embryo exposed to 0.501 mg/L; (**F**) 72 hpf embryo exposed to 5.01 mg/L; (**G**) 96 hpf embryo exposed to 5.01 mg/L; (**H**) 96 hpf embryo exposed to 50.1 mg/L; (**I**) 120 hpf embryo exposed to 0.501 mg/L; (**J**) 120 hpf embryo exposed to 5.01 mg/L. (**K**) 120 hpf embryo exposed to 0.1 mg/L of B(a)P; (**L**) 120 hpf embryo exposed to 0.5 mg/L of B(a)P. Scale bars: 200 µm.

The 48 hpf embryos exposed to 6.9 mg/L of 50 nm NPs or 500 nm NPs did not show perceptible plastic particles over surface of the chorion, allowing observation of the embryo development (Figure S2A,B), while 48 hpf embryos exposed to a comparable concentration of 4.5 µm MPs (5.01 mg/L) presented a fully covered chorion surface (Figure S2C). This full coverage of the chorion resulted in a rare case of a 120 hpf unhatched embryo exposed to 5.01 mg/L of 4.5 µm MPs-B(a)P that was still alive at the end of the experiment (Figure S2D). Zebrafish embryos exposed to NPs and MPs with sorbed B(a)P were also observed under the confocal microscope (Figure 5 and Figure 6). Unexposed embryos did not show fluorescence at any time (Figure 5A,D,H,K). The 24 and 48 hpf zebrafish embryos exposed to 500 nm NPs-B(a)P showed fluorescence on the chorion surface at exposure concentrations of 0.687 mg/L (Figure 5B,C,E) and of 6.87 mg/L. Only at the highest exposure concentration of 500 nm NPs-B(a)P was fluorescence detected in the yolk sac of 24 and 48 hpf zebrafish embryos (Figure 5C,F,G). For the highest concentration of 500 nm NPs-B(a)P (6.87 mg/L), the fluorescence continued to be present in 72 hpf hatched embryos (Figure 5I), with an intense fluorescence in the yolk sac. In 96 hpf embryos, similar localization of fluorescence was observed at 0.687 mg/L of 500 nm NPs-B(a)P (Figure 5J). In addition, a fluorescence signal around the embryo eye was observed. At the end of the exposure, fluorescence was detected in the 120 hpf embryos exposed to above 0.069 mg/L of 500 nm NPs-B(a)P (Figure 5L).

Fluorescence due to the exposure to 4.5 µm MPs-B(a)P (Figure 6) was first detected in 24 hpf embryos exposed at concentrations higher than 5.01 mg/L (Figure 6A,B). At 5.01 mg/L, fluorescence was only detected over the embryo chorion (Figure 6A), and, at the highest exposure concentration, it was also detected in the yolk sac (Figure 6B). The same localization of the fluorescence was observed in 48 hpf embryos exposed to 5.01 mg/L (Figure 6C), and in embryos exposed to 50.1 mg/L (Figure 6D) a more intense fluorescence was observed, compared to 24 hpf embryo exposed to the same concentration. The 72 hpf zebrafish embryos exposed to 4.5 µm MPs-B(a)P (Figure 6E,F) showed fluorescence in the yolk sac at exposure concentrations above 0.501 mg/L. A similar signal in the yolk sac was observed in 96 hpf zebrafish embryos exposed to 5.01 and 50.1 mg/L of 4.5 µm MPs-B(a)P (Figure 6G,H). The 120 hpf embryos presented fluorescence at exposure concentrations higher than 0.501 mg/L of 4.5 µm MPs-B(a)P (Figure 6I) and up to 5.01 mg/L (Figure 6J). As it can be seen, the fluorescence intensity was higher for zebrafish embryos exposed to 500 nm NPs-B(a)P (Figure 5) than to 4.5 µm MPs-B(a)P (Figure 6). The 120 hpf zebrafish embryos exposed to different B(a)P concentrations (0.1 and 0.5 mg/L, Figure 6K,L) presented fluorescence with a similar intensity to embryos exposed to MPs contaminated with B(a)P.

## 4. Discussion

In the present study, we aimed to assess the potential acute toxicity and bioavailability of PS NPs and MPs in brine shrimp larvae and zebrafish embryo development and whether these plastic particles can act as carriers of PAHs. Three plastic sizes were used, in order to investigate the effect of this parameter on their toxicity and bioavailability. Commercial PS NPs and MPs were chosen for this study, due to their availability in different and homogeneous sizes, which makes them very suitable for studying size-dependent effects, and due to their widespread use in laboratory experiments [41], and in spite of their lower environmental relevance compared to other polymers such as PP or PVC [42,43] or environmental NPs and MPs [44]. Especially in the case of Fluoresbrite^®^ 50 nm NPs, which, according to the manufacturer’s information, are designed as standards for instrument calibration and probes for cellular studies, fluorescent labelling greatly facilitates their localization and distribution within the organisms by non-invasive microscopical techniques, without interfering in the individuals’ development.

Previous studies that tested the toxicity of PS NPs and MPs in different species of brine shrimps reported no significant effects on survival [8,9,12,45,46]. In *Artemia parthenogenetica*, acute effects were not observed after 14 days of exposure to low concentrations of 10 µm PS MPs (1–1000 particles/mL or 0.55–550 µg/L). However, alterations at cell level, such as abnormal ultrastructure of intestinal epithelial cells and appearance of autophagosomes, which could affect the energetic system, were described [9]. Similar results were obtained in *Artemia franciscana*. Exposure to 100 nm PS NPs did not cause acute toxicity with a LC_50_ value higher than the highest tested concentration (100 mg/L) [12]. For smaller plastics, such as functionalized PS NPs (40 nm PS-COOH and PS-NH2), toxicity tests were performed in *A. franciscana* larvae, and no effect was recorded on mortality, even for the highest exposure concentration (100 mg/L) [8]. Nevertheless, recent studies indicated that other polymers, such as PP can exert more toxic effects on brine shrimps. Jeyavani and coworkers reported an increased oxidative burst and mortality (LC_50_ 40.95 mg/L) in nauplii exposed to 11.86–44.62 µm PP MPs [11]. In the present study, LC_50_ values higher than the highest tested concentrations, 50.1 mg/L for 4.5 µm PS MPs and 6.9 mg/L for 50 nm and 500 nm PS NPs, were estimated, in agreement with the previously mentioned results reported in the literature.

Survival rate, hatching time, and malformation prevalence in zebrafish embryos exposed to the different sized MPs and to NPs did not show any significant change compared to the control values. Acute effects in zebrafish embryos have not been reported in the literature, and sublethal effects have not always been reported [19,21,22,47,48]. Brun et al. [22] investigated the potential toxicity of PS NPs (25 nm) on the immune system of the skin and intestine of zebrafish embryos after injection of 1 nL of 1 mg NPs/L at 30 hpf. NP injection did not provoke significant changes in the survival or hatching rate of 54 hpf zebrafish embryos, but upregulation of immune system-related genes (*interleukin1β* and *chemokine (C-C motif) ligand 20a*) was observed. Karami et al. [21] exposed zebrafish embryos to a mixture of low-density PE fragments (5–500 µg/L) of different sizes (<17.6 µm). No effect was observed after 10 days of exposure for the assessed genes (*casp8, casp3a, sod1, gstp1, and cat*), while a significant downregulation of *cat*, *casp3a,* and *casp9* after 20 days of exposure, which resulted in minimal impact on the organisms, was reported. Nevertheless, effects on swimming mobility of zebrafish embryos after exposure to 1 mg/L of 1 µm PS MPs were observed, without significant changes in hatching rate, compared to controls [19]. In addition, upregulation of inflammation- (*interleukin1β*) and oxidative stress-related genes (*catalase*) in embryos exposed to 1 mg/L of PS MPs was observed. Thus, overall, NPs and MPs are reported as non-acutely toxic materials.

NPs and non-contaminated MPs did not cause acute toxic effects, but the exposure of early life stages of brine shrimps to NPs and MPs with sorbed B(a)P provoked significant effects on survival. The 500 nm NPs-B(a)P were toxic for 24 hph brine shrimp larvae after 48 h of exposure with a LC_50_ value of 4.75 mg/L. To the best of our knowledge, these effects of NPs associated with POPs on brine shrimp larvae have not been previously reported. In *D. magna*, another aquatic branchiopod commonly used for aquatic toxicity assessment, higher lethality was recorded in individuals co-exposed for 48 h to PS NPs (100 nm) and PCBs (0.64 mg/L) than in those exposed to PCBs alone. The combined toxicity to *D. magna* depended on the relative concentration of NPs and PCBs, with PCBs being less toxic when combined with low concentrations of NPs (<1 mg/L), while higher NP concentrations enhanced the lethality [31]. Waterborne exposure of *D. magna* to larger PS NPs of up to 1 µm (3 × 10^5^ particles/mL or 0.038 mg/L), in combination with previously known toxic concentrations of the insecticides dimethoate and deltamethrin, did not lead to any effect on the studied endpoints, such as survival and mobility [49]. After being internalized, B(a)P sorbed to the plastic particles could be released to the brine shrimp digestive system, increasing its toxicity [50]. In the present study, significant differences were found between the exposure of brine shrimp larvae to a similar concentration of 4.5 µm MPs-B(a)P (5.01 mg/L) and of 500 nm NPs-B(a)P (6.87 mg/L), showing that the risk of death increased with the decrease of contaminated plastic particle size. Thus, results from the literature and from the present study suggest that in the case of contaminated NPs and MPs, size plays a critical role in toxicity, with smaller plastic particles being more toxic for aquatic organisms than larger ones, when combined with other toxic pollutants. This seems to be due to the higher surface/volume ratio of smaller particles, which confers them a higher capacity to carry B(a)P [29].

Previous studies have addressed the potential toxicity of NPs and MPs combined with organic compounds on zebrafish development [20,33,51]. In zebrafish embryos of 3 hpf, co-exposure for 48 h and 72 h to 45 µm PS MPs or 50 nm PS NPs with EE2 (2 µg/L and 20 µg/L) produced an increase of toxicity at the highest EE2 concentration, showing an increase in catalase (CAT) activity and glutathione (GSH) content compared to control embryos. A high concentration of EE2 combined with MPs was, in terms of oxidative stress, more toxic than a high concentration of EE2 combined with NPs, showing that the bioavailability of EE2 was higher in the presence of MPs than of NPs [20]. On the contrary, co-exposure to 200–250 μm PVC MPs and EE2 or phenanthrene reduced the bioavailability of the organic compounds to zebrafish embryos, resulting in no effects for the tested treatments [33]. The intermediate MP size (11–13 µm PE, 100 mg/L) spiked with B(a)P (16.87 µg/g) provoked an increase of biotransformation metabolism (EROD activity and *cytochrome P450 1A* (*cyp1a*) transcription levels), indicating the successful transfer of B(a)P via MPs to the embryos, but not acute embryotoxicity [51]. In the present study, only exposure to 4.5 µm MPs with sorbed B(a)P caused toxicity to 120 hpf embryos (EC_50_ = 45.57 ± 9.12 mg/L or 9.1 × 10^6^ particles/mL), while no significant effects were recorded, even at the highest tested concentration of 500 nm NPs-B(a)P (EC_50_ > 6.87 mg/L or 10^8^ particles/mL). The type of malformations observed in 120 hpf zebrafish embryos exposed to 4.5 µm MPs-B(a)P were similar to those provoked by B(a)P exposure. With 30% of the embryos exposed to 52.5 mg/L of 4.5 µm MPs-B(a)P presenting pericardial edema. As previously mentioned, cardiotoxicity is a common effect of PAHs from crude oil in zebrafish embryos [52].

B(a)P is a well-known toxic compound for aquatic organisms [53,54,55]. In this study, estimated LC_50_ values for 48 hph brine shrimp larvae exposed for 24 and 48 h were 0.004 ± 0.197 mg/L and 0.002 ± 0.119 mg/L, respectively, whereas the EC_50_ value, based on malformation appearance, for zebrafish embryo exposed for 120 h was 3.55 ± 0.68 mg/L. Brine shrimps presented an increased sensitivity throughout the developmental stages (24 hph to 96 hph) when exposed to B(a)P. This increased sensitivity could be due to the progressive loss of energy reservoirs present in the first developmental stages and the lack of feeding during the assay. The LC_50_ values for 48 hph brine shrimp larvae exposed to B(a)P were similar to those reported for *D. magna*. Exposure of *D. magna* for 48 h to 1–32 µg/L B(a)P without feeding resulted in a LC_50_ value of 4.7 µg/L [55]. PAHs, together with other organic compounds, such as PCBs, are amongst the most toxic compounds to planktonic crustaceans (branchiopod, copepod, and ostracod), primarily due to the higher toxicity and greater persistence of their metabolites, than of the parent compounds [54]. The observed mortality in early stages of brine shrimps at low B(a)P exposure concentrations shows the sensitivity of this organism to PAH exposure; being a suitable model for toxicity evaluation of these compounds.

Zebrafish embryos were less sensitive to B(a)P exposure than brine shrimp larvae. Nevertheless, B(a)P has also been proven to be a toxic PAH for zebrafish embryo development [56,57,58,59]. Knecht et al. [58] observed a non-significant increase of mortality rate and malformation prevalence in 120 hpf zebrafish embryos exposed to 0.1 and 1 mg/L of B(a)P (1% DMSO), but the alteration of the swimming behavior resulted in hyperactivity of embryos exposed to B(a)P. Furthermore, increasing B(a)P concentration up to 2.5 mg/L caused mortality in 72 hpf zebrafish embryos, with a LC_50_ value of 1.285 mg/L and a EC_50_ for malformations of 0.131 mg/L [56]. A higher B(a)P concentration (500 µM = 12.6 mg/L) provoked 40–50% of malformed embryos (72 hpf) without further changes in their heart rate, but oxidative damage to DNA was observed in 72 hpf zebrafish embryos exposed to a lower B(a)P concentration (1 µM = 0.25 mg/L) [57]. Exposure to 1 µM B(a)P also caused genotoxicity in 96 hpf embryos [59]. The higher sensitivity observed for brine shrimps exposed to B(a)P than for zebrafish embryos was possibly due to the efficiency of aquatic vertebrates, even at the initial stages of development, for detoxification of organic compounds compared to invertebrates [60].

Previous works reported the use of B(a)P concentrations ranging from 0.2 to 25.2 mg/L for developmental and sublethal toxicity studies in zebrafish embryos [56,58]. The low solubility of B(a)P in water (1.64 µg/L) [61] makes it difficult to reach acutely toxic concentrations for organisms [62,63], which are usually above solubility point, even using DMSO as vehicle. Due to this low solubility, B(a)P in water tends to aggregate, leading to analytical deviations. The analytical results reported by Costa et al. [62] and Zhao et al. [63] showed that none of the B(a)P solutions reached the expected nominal concentration. Moreover, B(a)P is an hydrophobic compound that tends to be sorbed on the plastic material used for biological tests, especially on uncoated PS microplates [64,65]. According to Fischer et al. [65], exposure to PAHs with partition coefficients (log K_ow_) higher than 6, such as B(a)P, should be carefully monitored, especially for low concentrations, due to the predicted losses higher than 80% from initial concentration, as a result of adsorption to PS microplates. Chlebowski et al. [64] also reported a high sorption of different PAHs (fluoranthene, pyrene, chrysene, and B(a)P) at the highest exposure concentration used (0.32 µM of each PAH studied). In the case of B(a)P, 48% of the total (80.64 µg/L = 0.32 µM) remained in the water, 39% was sorbed onto the walls, and 13% was assimilated by the zebrafish embryos. In agreement, our results showed that the B(a)P concentration decreased when the test media were placed into the PS microplate wells, likely due to both, degradation, and sorption to the microplate walls, with the loss percentage being higher for the lower concentrations.

Confocal micrographs showed the abundant presence of 50 nm fluorescent NPs within the brine shrimp digestive tract. Non-fluorescently labeled 4.5 µm MPs were also easily observed in brine shrimp digestive tract, whereas 500 nm NPs were not distinguishable under the light microscope. Nevertheless, taking into account that 50 nm NPs and 4.5 µm MPs were ingested by brine shrimp, assimilation of 500 nm NPs was also expected to occur. Localization of plastic particles in the digestive tract of brine shrimp has been reported for NPs and MPs of different sizes [8,13]. For smaller plastics, such as 40 nm anionic carboxylated (PS-COOH) and 50 nm cationic amino (PS-NH2) fluorescent PS NPs, ingestion by *A. franciscana* was observed for concentrations ranging from 5 to 100 mg/L, although fluorescence was only detected on the surface of the feces of brine shrimp [8]. Ingestion was observed for a concentration ranging from 1 to 1000 particles/mL of 10 µm PS MPs by *A. parthenogenetica* (<24 h old) over 24 h [13]. As previously highlighted, it is evident that MPs and NPs are ingested by brine shrimps, but signs of internalization into tissues have not yet been reported. The ingestion of NPs and MPs by zooplanktonic species, such as brine shrimp, which are consumed by numerous organisms at higher levels of the trophic chain, is a potential risk for plastic transfer and accumulation in the food web, even between different zooplanktonic organisms [45,66] in aquatic ecosystems.

In the case of zebrafish, NPs spread through the body and are accumulated in specific organs (eye, yolk sac, and tail), even in early development stages, mainly due to the large pore size of the embryo chorion (600–700 nm) [67]. Using confocal microscopy, fluorescence was detected in several optical sections, indicating internalization of NPs in the eye, as also reported by van Pomeren et al. [68] for 25 and 50 nm PS NPs, but not for larger NPs (200 nm). Other nanomaterials, such as 27 nm SiO_2_ fluorescent nanoparticles have also been localized into zebrafish embryo eyes at 120 hpf [69]. The presence of nanoparticles in the yolk sac is usually reported for zebrafish embryos [23,41,69]. Zebrafish embryos exposed to fluorescent PS NPs presented a higher internalization of NPs with the increase of larval age [23,70], with fluorescence being located on the surface of the chorion, in the yolk sac (24 hpf), and in the head [23]. A different organ accumulation of NPs, in the pancreas and liver, was observed in 120 hpf zebrafish embryos [23]. In this study, a notable increase of fluorescence was observed near the tail and the yolk sac, in the form of irregular deposits. As seen by Evensen et al. [71], an injection of fluorescent PS NPs (100 nm) in the cardinal vein produced an accumulation of NPs on macrophages, with similar structure and distribution to those found in our study. A similar distribution of fluorescent PS NPs was observed at neuromast level, localized inside macrophages [22]. NPs can be a potential source of damage to nervous and digestive systems, due to their reported accessibility to different organs. The presence of NPs in the eyes and brain leads to, for example, locomotor alterations [20,72]. Thus, further studies on the long-term effects in juveniles and adults after an acute exposure event at embryo stage would be of great interest.

Ingestion or internalization of MPs or contaminated MPs was not observed in zebrafish embryos in the present study, but B(a)P fluorescence was observed all over the zebrafish embryos exposed to contaminated MPs, from very early stages. This fluorescence could be due to the uptake of B(a)P desorbed from the MPs to the medium or by direct contact of MP-B(a)P with the embryo’s surface.

Results for brine shrimp and zebrafish exposures showed a successful transport of B(a)P from MPs to the organisms, in a wide range of concentrations (0.00069 to 6.9 mg/L for 500 nm NPs and 0.501 to 50.1 mg/L for 4.5 µm MPs). In brine shrimps, the results of the exposures to 500 nm NPs and to 4.5 µm MPs-B(a)P differed. In the case of 500 nm NPs-B(a)P, fluorescence was only detected in the digestive tract and in the feces, while for 4.5 µm MPs-B(a)P, fluorescence was only observed all over the body and in the digestive tract. As shown by Batel et al. [73], 1–5 µm and 10–20 µm PE MPs-B(a)P released the B(a)P all over the body of brine shrimps and then B(a)P was transferred from brine shrimps to adult zebrafish via their diet. Due to the capacity of brine shrimps to filter large amounts of water, exposure to MPs with sorbed B(a)P can lead to increased exposure to B(a)P compared to other organisms that are more selective in terms of feeding, representing a risk to these organisms. In zebrafish embryos, B(a)P fluorescence was observed on the surface of the chorion and in the yolk sac. The successful transport of B(a)P into embryos by MPs of both sizes was observed, even through the chorion wall, proving the risk of MPs as vectors of other compounds. However, it was not possible to decipher whether the transport of B(a)P took place by desorption of B(a)P from MPs in the exposure media or by B(a)P desorbed after ingestion.

## 5. Conclusions

In summary, NPs and MPs alone did not cause acute effects in zebrafish embryos and brine shrimp larvae, even if they were ingested by both organisms. Toxic effects occurred when NPs (500 nm) and MPs (4.5 µm) were contaminated with B(a)P, and exposure to NPs and MPs with sorbed B(a)P resulted into B(a)P accumulation in brine shrimp larvae and zebrafish embryos. MP size played a significant role in explaining the toxicity of MPs with sorbed B(a)P, implying a potential risk for brine shrimps when MP size decreased, due to the increase of surface to volume ratio, which results in a higher quantity of B(a)P sorbed for potential transfer. Thus, polystyrene NPs and MPs of different size are likely to act as vectors of PAHs in the aquatic environment, modulating their bioavailability and provoking toxic effects in organisms that play important roles in ecosystems. However, a lot of work remains to be done, especially for standardization of MP experiments, as well as for the use of environmentally relevant MPs at realistic concentrations, in order to evaluate the threat posed by NP and MP contamination. Studies of the long-term consequences at physiological and behavioral levels are required.

## Figures and Tables

**Table 1:** Relationship between the plastic particle concentrations, in terms of mass and in terms of number of particles for the three sizes used.

50 nm NPs		500 nm NPs		4.5 µm MPs	
Particles/mL	mg/L	Particles/mL	mg/L	Particles/mL	mg/L
10^6^	0.000069				
		5 × 10^3^	0.00034		
10^7^	0.00069		0.00069		
				5 × 10^2^	0.025
10^9^	0.069	10^6^	0.069	10^3^	0.069
10^10^	0.687	10^7^	0.687	10^7^	0.687
10^11^	6.87	10^8^	6.87	10^8^	6.87
				10^6^	50.1

**Table 2:** B(a)P concentration (mg/L) over time for the five exposure concentrations. Percentage of recovered B(a)P compared to that measured at time 0 is indicated in brackets. Values are given as mean ± S.D. (*n* = 2).

Nominal Concentration	Measured Concentration			
	0 h	24 h	48 h	120 h
0.1	0.029 ± 0.005	0.029 ± 0.006 (100%)	0.022 ± 0.002 (75.9%)	0.014 ± 0.001 (48.3%)
0.5	0.315 ± 0.142	0.432 ± 0.084 (137.1%)	0.2578 ± 0.011 (81.8%)	0.282 ± 0.012 (89.5%)
1	0.496 ± 0.111	0.503 ± 0.006 (101.4%)	0.301 ± 0.001 (60.7%)	0.265 ± 0.025 (53.4%)
5	1.496 ± 0.707	1.173 ± 0.505 (78.4%)	0.738 ± 0.299 (49.3%)	1.118 ± 0.053 (74.7%)
10	4.922 ± 1.995	5.023 ± 0.397 (102.1%)	4.927 ± 0.283 (100.1%)	5.269 ± 1.095 (107%)

**Table 3:** Effects on survival (percentage over the whole sample, *n* = 30 individuals) of the 24 h and 48 h exposures to NPs and to MPs alone and with sorbed B(a)P of 24 hph and 48 hph brine shrimp larvae.

	Concentration (mg/L)	24 hph Larvae		48 hph Larvae	
		24 h	48 h	24 h	48 h
50 nm NPs	0	100.0	96.8	93.8	100.0
	0.000069	93.3	90.0	100.0	100.0
	0.00069	97.0	97.0	96.8	93.3
	0.069	96.9	96.9	93.3	100.0
	0.687	96.7	90.0	100.0	100.0
	6.87	93.1	89.7	100.0	93.3
500 nm NPs	0	96.8	93.5	100.0	100.0
	0.00034	93.5	96.8	100.0	96.8
	0.00069	93.3	93.3	100.0	100.0
	0.069	96.8	87.1	100.0	91.2
	0.687	90.9	87.9	100.0	97.0
	6.87	97.1	94.3	100.0	96.9
4.5 µm MPs	0	100.0	96.8	100.0	93.3
	0.0251	100.0	96.8	100.0	96.8
	0.0501	96.8	93.5	100.0	80.0
	0.501	100.0	93.3	96.9	93.8
	5.01	96.7	93.3	96.7	87.1
	50.1	100.0	96.7	100.0	75.0
500 nm NPs-B(a)P	0	96.7	86.7	100.0	80.0
	0.00034	93.3	80.0 *	93.3	53.3 *$
	0.00069	100.0	60.0 *$	100.0	60.0
	0.069	96.7	83.3	100.0	56.7
	0.687	100.0	73.3	100.0	56.7 $#
	6.87	93.3	36.7 *$#	100.0	60.0 $#
4.5 µm MPs-B(a)P	0	96.7	76.7	93.3	80.0
	0.0251	90.0	60.0 $	96.7	90.0
	0.0501	100.0	83.3	100.0	86.7
	0.501	86.7	56.7 $	100.0	90.0
	5.01	90.0	70.0	100.0	70.0
	50.1	100.0	63.3 $	93.3	90.0
B(a)P	0	97.2	91.7	93.3	80.0
	0.1	100.0	65.6 *	0.0	0.0
	0.5	94.3	51.4 *	0.0	0.0
	1	97.0	57.6 *	0.0	0.0
	5	87.9	45.5 *	0.0	0.0
	10	90.6	59.4 *	0.0	0.0

Asterisks (*) indicate statistically significant differences (*p* < 0.05) compared to the control group according to the binomial logistic regression. Dollars ($) indicate statistically significant differences (*p* < 0.05) compared to the exposure to NPs and MPs of the same size without B(a)P at the same concentration. Hashes (#) indicate statistically significant differences (*p* < 0.05) between 500 nm NPs-B(a)P and 4.5 µm MPs-B(a)P at the same exposure concentration. The corresponding odd ratios and confidence intervals are given in Table S2.

**Table 4:** Results of the developmental parameters of zebrafish embryos exposed to NPs and to MPs alone and in combination with B(a)P. Data on survival and malformation prevalence at 120 hpf are given as percentages of the whole sample (*n* = 30–36 individuals). Data on hatching time are shown as means ± standard deviations.

	Exposure Conc. (mg/L)	Surv. (%)	Hatching Time (h)	Malform. (%)	Type of Malform. (%)			
					SC	PE	YE	EA
50 nm NPs	0	97.2	72	11.4	11.4	0	0	0
	0.000069	96.7	72	20.7	20.7	0	0	0
	0.00069	100	72	6.7	6.7	0	0	0
	0.069	93.3	72	7.1	3.6	3.6	0	0
	0.687	96.7	72	10.3	10.3	0	0	0
	6.87	90	72	3.7	3.7	0	0	0
500 nm NPs	0	94.4	72	8.8	8.8	0	0	0
	0.00034	93.3	72	10.7	10.7	0	0	0
	0.00069	90	71.1 ± 4.6	11.1	11.1	0	0	0
	0.069	96.7	72	17.2	17.2	0	0	0
	0.687	96.7	72	20.7	20.7	3.5	3.5	3.5
	6.87	93.3	72	21.4	14.3	10.7	0	0
4.5 µm MPs	0	94.4	71.3 ± 4.1	2.9	2.9	0	0	0
	0.0251	96.7	72	10.3	6.9	3.4	0	0
	0.0501	100	72	16.7	10	6.7	0	0
	0.501	93.3	70.3 ± 6.3	7.1	7.1	0	0	0
	5.01	100	72	16.7	13.3	3.3	0	0
	50.1	100	70.4 ± 6.1	20	16.6	6.7	0	0
500 nm NPs-B(a)P	0	94.4	58.6 ± 12.1	16.7	13.9	2.8	0	0
	0.00034	93.3	55.7 ± 11.4	16.7	10	6.7	0	0
	0.00069	90	54.2 ± 10.7	26.7	26.7	6.6	3.3	0
	0.069	96.7	57.1 ± 11.9	16.7	13.3	3.3	0	0
	0.687	96.7	55.4 ± 11.3	16.7	10	6.7	0	0
	6.87	93.3	56.6 ± 12.0	10	6.7	6.7	0	0
4.5 µm MPs-B(a)P	0	100	71.3 ± 4	11.1	11.1	0	0	0
	0.0251	100	72	16.7	16.7	0	0	0
	0.0501	100	72.0	16.7	16.7	3.3	0	0
	0.501	100	70.4 ± 6.1	13.3	10	3.3	0	0
	5.01	100	72	26.7	20	10	3.3	0
	50.1	100	70.4 ± 6.1	56.7 *$	36.7	30	6.7	0
B(a)P	0	97.2	67.2 ± 9.7	11.6	8.6	2.9	0	0
	0.1	96.7	69.5 ± 8.3	13.8	13.8	0	0	0
	0.5	86.7	69.6 ± 7.8	24.1	23.1	7.7	0	0
	1	86.7	69.2 ± 7.8	26.9	23.1	7.7	3.8	0
	5	96.7	70.3 ± 6.2	50 *	27.5	20.6	3.4	0
	10	96.7	70.3 ± 6.2	58.6 *	44.8	24.1	0	0

Conc. = concentration; EA: eye abnormality; Malform. = malformation; PE = pericardial edema; SC = spinalcord flexure; Surv. = survival; YE = yolk sac edema. Asterisks (*) indicate statistically significant differences (*p* < 0.05) compared to the control group according to the binomial logistic regression. Dollars ($) indicate statistically significant differences (*p* < 0.05) compared to the exposure to NPs and MPs of the same size without B(a)P at the same exposure concentration. The values of the corresponding odd ratios and confidence intervals are given in Table S4.

## Data Availability

Data are contained within the article.

## Supplementary Materials

The following supporting information can be downloaded at: https://www.mdpi.com/article/10.3390/nano12060941/s1, Figure S1: Measured concentrations from a nominal concentration of 2.51 mg/L or 5 × 10^4^ particles/mL of 4.5 µm MPs using a cell counter before and after filtration using a polyethersulfone filter (0.45 µm filter pore). Bars represent the mean of three instrumental replicates with their corresponding standard deviations. The percentage of MPs measured from the nominal concentration is given above each bar. Figure S2. Micrographs of zebrafish embryos exposed to similar masses of plastics of different sizes. (A) 48 hpf embryo exposed to 6.87 mg/L of 50 nm NPs; (B) 48 hpf embryo exposed to 6.87 mg/L of 500 nm NPs; (C) 48 hpf embryo exposed to 5.01 mg/L of 4.5 µm MPs; (D) non-hatched 120 hpf alive embryo exposed to 5.01 mg/L of 4.5 µm MPs-B(a)P. Scale bars: 100 µm. Table S1: Effect on survival (%) of the exposure of 24 hph and 48 hph brine shrimp larvae to DMSO for 24 h and 48 h. Table S2: Odd ratio values indicating the risk of death (immobilization) for brine shrimp larvae exposed to NPs and MPs alone or in combination with B(a)P. Table S3: Odd ratio values indicating the risk of malformation in 120 hpf zebrafish embryos exposed to 4.5 µm MPs alone or in combination with B(a)P or to B(a)P alone. Table S4: Effects of 120 h DMSO exposure on developmental parameters of zebrafish embryos.
