# Supplementary material for: Screening of the Toxicity of Polystyrene Nano- and Microplastics Alone and in Combination with Benzo(a)pyrene in Brine Shrimp Larvae and Zebrafish Embryos
Source: Nanomaterials (Basel). 2022 Mar 12;12(6):941. doi: 10.3390/nano12060941 (PMC8948799; doi:10.3390/nano12060941)
Supplement: Supplementary file 1 [file nanomaterials-12-00941-s001.zip › nanomaterials-1600401-supplementary.pdf]

nanomaterials

Supplementary Materials

Screening of the Toxicity of Polystyrene Nano- and Microplastics Alone and in Combination with Benzo(a)pyrene in Brine Shrimp Larvae and Zebrafish Embryos

Ignacio Martínez-Álvarez [1,2], Karyn Le Menach [2], Marie-Hélène Devier [2], Miren P. Cajaraville [1], Hélène Budzinski [2] and Amaia Orbea [1,*]

[1] CBET Research Group, Department of Zoology and Animal Cell Biology, Research Centre for Experimental Marine Biology and Biotechnology PiE and Science and Technology Faculty, University of the Basque Country (UPV/EHU), E-48940 Leioa, Spain; ignacio.martinez@ehu.eus (I.M.-Á.); mirenp.cajaraville@ehu.eus (M.P.C.)
[2] University of Bordeaux, UMR 5805 CNRS, EPOC, Laboratory of Physico- and Toxico-Chemistry of the Environment, F-33405, Talence Cedex, France.
karyn.le-menach@u-bordeaux.fr (K.L.M.); marie-helene.devier@u-bordeaux.fr (M.-H.D.); helene.budzinski@u-bordeaux.fr (H.B.)
* Correspondence: amaia.orbea@ehu.eus; Tel.: +34-946-012-735

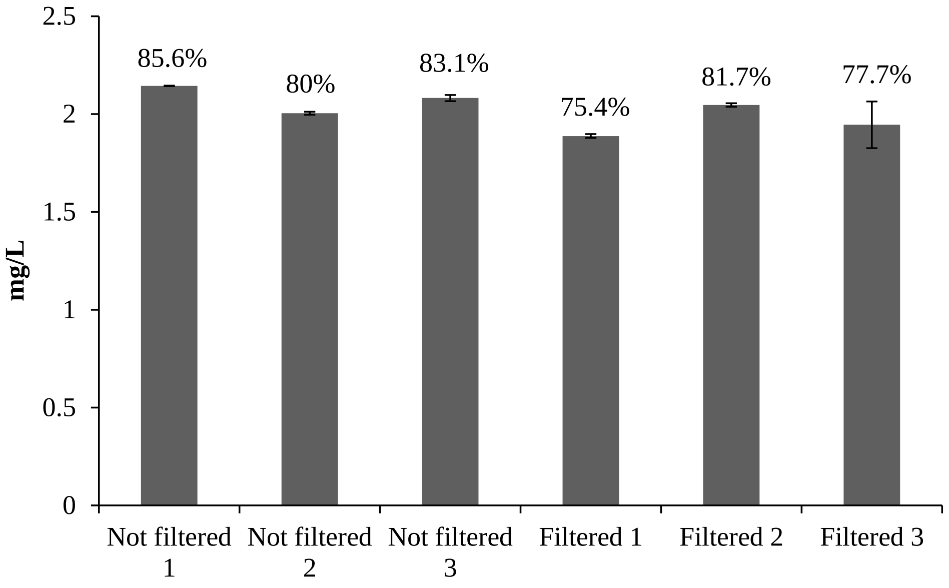

Figure S1- Measured concentration from a nominal concentration of 2.51 mg/L or 5.10^4 particles/mL of 4.5 µm MPs using a cell counter before and after filtration using a polyethersulfone filter (0.45 µm filter pore). Bars represent the mean of 3 instrumental replicates with their corresponding standard deviation. The percentage of MP measured from the nominal concentration is given above each bar.

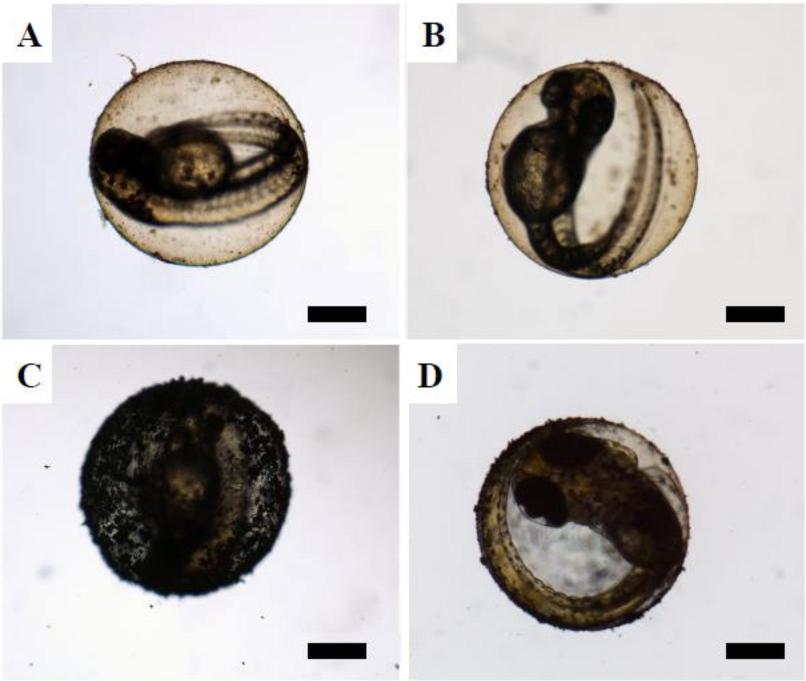

Figure S2. Micrographs of zebrafish embryos exposed to similar masses of plastics of different sizes. A) 48 hpf embryo exposed to 6.87 mg/L of 50 nm NPs; B) 48 hpf embryo exposed to 6.87 mg/L of 500 nm NPs; C) 48 hpf embryo exposed to 5.01 mg/L of 4.5 µm MPs; D) non-hatched 120 hpf alive embryo exposed to 5.01 mg/L of 4.5 µm MPs-B(a)P. Scale bars: 100 µm.

Table S1- Effect on survival (%) of the exposure of 24 hph and 48 hph brine shrimp larvae to DMSO for 24 h and 48 h.

Concentration (v/v)	24 hph		48 hph	
	24 h	48 h	24 h	48 h
0	100	97	100	97
0.01%	97	93	97	93
0.10%	100	93	100	100

nanomaterials

Table S2- Odd ratio values indicating the risk of death (immobilisation) for brine shrimp larvae exposed to MPs alone or in combination with B(a)P.

Treatment test		Treatment for comparison	Conc. (mg/L)	Odd ratio	Confidence interval (5%, 95%)	*p* value
500 nm NPs-B(a)P	24 hph 48 h	Control	0.00034	1.94 10^{54}	7.83 10^{50} - 4.78 1057	0
			0.00069	1.94 10^{54}	7.83 10^{50} - 2.71 10^{271}	0
			6.87	1.422	1.179 - 1.715	0.000
	48 hph 48 h	Control	0.00034	1.94 10^{54}	7.83 10^{50} - 2.72 10^{271}	0
B(a)P	24 hph 48 h	Control	0.1	2.82 10^{8}	25.257 - 3.16 10^{15}	0.008
			0.5	196	7.939 - 4838.811	0.000
			1	10.706	2.148 - 53.348	0.001
			5	1.741	1.263 - 2.399	0.000
			10	1.250	1.064 - 1.469	0.002
500 nm NPs-B(a)P	24 hph 48 h	500 nm NPs	0.00069	9.333	1.866 - 46.683	0.001
			6.87	24.182	4.808 - 121.625	0.000
	48 hph 48 h	500 nm NPs	0.00034	25.375	3.050-211.104	0.000
			0.687	22.176	2.661-184.798	0.000
			6.87	19.333	2.313-161.565	0.000
4.5 µm MPs-B(a)P	24 hph 48 h	4.5 µm MPs	0.025	19.333	2.313 - 161.565	0.000
			0.501	10.706	2.148 - 53.348	0.001
			50.1	16.789	2.001 - 140.898	0.001
4.5 µm MPs-B(a)P	24 hph 48 h	500 nm NPs-B(a)P	5.01, 6.87	0.706	0.539 - 0.924	0.009
	48 hph 48 h	500 nm NPs-B(a)P	0.501, 0.687	0.617	0.436 - 0.875	0.003
			5.01, 6.87	0.639	0.450 - 0.906	0.006

Table S3- Odd ratio values indicating the risk of malformation in 120 hpf zebrafish embryos exposed to 4.5 µm MPs alone or in combination with B(a)P or to B(a)P alone.

Treatment test	Treatment for comparison	Conc. (mg/L)	Odd ratio	Confidence interval (5%, 95%)	*p* value
4.5 µm MPs-B(a)P	Control	50.1	1.045	1.019 - 1.072	0.0001
B(a)P	Control	5	1.373	1.081 - 1.744	0.0063
		10	1.239	1.098 - 1.397	0.0002
4.5 µm MPs-B(a)P	4.5 µm MPs	50.1	5.71	1.82 - 20.66	0.0000

Table S4. Effects of 120 h DMSO exposure on developmental parameters of zebrafish embryos.

Concentration (%, v/v)	% survival	hatching time (h)	% malformed embryos
0.0	100	70.0	8.3
0.01	100	68.7	10.0
0.1	100	69.3	13.3
